# Lateral flow assay as radiological prognosis factor of pulmonary cryptococcosis: a single center retrospective study in China

**DOI:** 10.3389/fcimb.2024.1497082

**Published:** 2025-01-21

**Authors:** Jiejun Shi, Jianhua Chen, Qianjiang Ding, Guoqing Qian, Zeqin Zhang, Qifa Song

**Affiliations:** ^1^ Department of Infectious Diseases, The First Affiliated Hospital of Ningbo University, Ningbo, Zhejiang, China; ^2^ Department of Radiology, The First Affiliated Hospital of Ningbo University, Ningbo, Zhejiang, China; ^3^ Department of Cardiology, The First Affiliated Hospital of Ningbo University, Ningbo, Zhejiang, China; ^4^ Medical Data Center, The First Affiliated Hospital of Ningbo University, Ningbo, Zhejiang, China

**Keywords:** pulmonary cryptococcosis, lateral flow assay, therapeutic efficacy, radiological prognosis, lesion distribution, HIV-negative

## Abstract

**Background:**

Lateral flow assay (LFA) has demonstrated high sensitivity and specificity for diagnosing cryptococcosis. However, its role in predicting therapeutic efficacy for pulmonary cryptococcosis (PC) remains underexplored.

**Methods:**

We conducted a retrospective analysis of HIV-negative patients with PC to describe the clinical profile and identify potential predictors of radiological prognosis.

**Results:**

All the 168 participants received antifungal therapy with a triazole agent. Of these, 84.5% experienced partial or complete absorption of pulmonary lesions. The results of the gamma test, chi-square trend test, and ordinal logistic regression all indicated that both baseline LFA and changes in LFA after treatment were significant predictors of imaging prognosis. The degree of radiological improvement was inversely associated with the baseline LFA positive grade(P for linear-by-linear association: 0.011, Spearman correlation coefficient = -0.17; γ= -0.368, P = 0.045). Patients with a decrease in LFA after therapy had significantly better radiological outcomes compared to those with equal or increased LFA(linear-by-linear association, P = 0.014, Spearman correlation coefficient = 0.188; γ = 0.371, P = 0.012). Additionally, favorable outcomes were more likely in patients with lesions confined to the right lung.

**Conclusions:**

LFA shows potential of monitoring radiological outcomes in pulmonary cryptococcosis.

## 1 Introduction

Although the global incidence of HIV-related cryptococcal meningitis has decreased during the past decade, cryptococcal infection remains the leading cause of death in patients with Acquired Immune Deficiency Syndrome (AIDS), accounting for one in five AIDS-related deaths all around the world (Tugume et al., 2023). It is estimated that the global burden of cryptococcal meningitis is approximately 152,000 cases per year, resulting in 112,000 deaths (Rajasingham et al., 2022). Intriguingly, compared to HIV-related individuals with cryptococcal meningitis (CM), non-HIV hosts have been reported to have a significantly higher mortality rate at 90 days (6.3% vs. 25.4% P = 0.0002), highlighting the need for more attention to immunocompetent cryptococcosis (Namie et al., 2024). Pulmonary cryptococcal infection is the second most common manifestation of cryptococcosis and is more frequently observed in immunocompetent patients (Allena et al., 2023; Shi et al., 2023). In contrast to CM, pulmonary cryptococcosis (PC) remains underdiagnosed, largely due to the lack of reliable diagnostic tools (Setianingrum et al., 2019). Moreover, the assessing therapeutic efficacy in PC is challenging. The radiological characteristics of PC are highly variable and can be easily misdiagnosed as bacterial pneumonia, lung tumor, tuberculosis, or other fungal infections, with nodules being the most commonly observed finding (Setianingrum et al., 2019).

Cryptococcal antigen (CrAg) tests, including lateral flow assay (LFA), latex agglutination (LA) test and cryptococcal glucuronoxylomannan antigen test, are pivotal to differentiate PC from other diseases with similar radiological manifestations. Although the CrAg test is generally considered less effective in diagnosing cryptococcosis with localized pulmonary involvement due to its high false negative rate (Zhu et al., 2018; Cheng et al., 2021), our previous research indicated that the extent of pulmonary lesions may influence LFA grade (Spearman r = 0.268, p<0.01) with most false-negative results occurring in cases of solitary nodules. Compared to LA, LFA showed higher sensitivity in diagnosing cryptococcosis, especially for those with localized pulmonary disease (Hevey et al., 2020). However, there have been few reports on the relationship between LFA and the radiological outcomes in PC following treatment. Therefore, we conducted a retrospective study to investigate the issues mentioned above.

## 2 Materials and methods

### 2.1 Study design and participants

This retrospective study was based on the data from the YIDUCLOUD platform of the First Affiliated Hospital of Ningbo University. Using the intelligent search engine on the Data Process & Application Platform, we identified 511 patients admitted to our hospital between January 2010 and January 2024, with a discharge diagnosis of PC. After reviewing their hospital records, we selected 168 eligible participants based on the following inclusion and exclusion criteria.

Inclusion criteria:

1. Confirmed diagnosis of cryptococcosis (Donnelly et al., 2020) 2. Newly developed pulmonary lesions unresponsive to antibacterial treatment 3. Age > 18 years old

Exclusion criteria:

1. Co-infected with HIV 2. cryptococcal meningitis or disseminated extrapulmonary cryptococcosis. 3. Incomplete clinical or laboratory data 4. Surgical resection of pulmonary lesions.

Participants with at least one of the following conditions were classified as immunocompromised: malignancy, autoimmune disease, chronic viral hepatitis B, diabetes, chronic kidney disease, tuberculosis, or long-term use of immunosuppressants.

### 2.2 Lateral flow assay

In the current study, LFA was performed on serum samples to assess cryptococcal capsular polysaccharide antigen using the Cryptococcus antigen detection kit (IMMY, USA). Results were read 10 minutes after sample addition, and LFA grades were classified as negative, weak positive or positive. A “weak positive” result indicates a band with lighter color than the positive band but deeper than the negative band. The antigen detection kit protocol provides detailed information on the operation and results. The term “baseline LFA” refers to the serum LFA test conducted within one week prior to the diagnosis of PC, while “post-treatment LFA” refers to the follow-up result of the serum sample within 5-7 months after the initiation of antifungal therapy. The changes in LFA were categorized as decreased or not decreased (equal or increased) based on the difference between baseline LFA and post-treatment LFA results.

### 2.3 Radiological examinations

All subjects underwent at least twice chest computed tomography (CT) scans throughout the whole therapy period. Regular CT scans were conducted using a variety of CT systems (Somatom Sensation 16, Siemens Medical Systems, Forchheim, Germany; Brilliance 16, Philips Medical Systems, Amsterdam, Netherlands; Aquilion 64, Toshiba Medical Systems, Otawara, Japan). 2 to 5 mm thick consecutive sections were taken from the lung apex to the base. The lung parenchyma was viewed using a window width of 1400–1600 Hu and a level of –550 to –600 Hu, while the soft tissue window was set at a width of 400 Hu and a level of 40 Hu. The chest CT scans were independently reviewed by two radiologists. In the case of discrepancies, a consensus decision was reached. The radiological outcome was categorized as aggravated, unchanged, partial absorption, or complete absorption, based on the comparison of chest CT images taken before and after treatment. The regression of lesions is comprehensively evaluated according to the change in area, location and radiodensity. Lesions with concurrent absorption and deterioration were classified as unchanged as well. The radiological morphology and extent were evaluated before antifungal therapy. The extent of pulmonary lesions was categorized as restricted lesions in a single lung lobe, diffused lesions in the unilateral lung, or diffused lesions in bilateral lungs. Masses refer to lesions with a diameter exceeding 3 cm while nodules were defined as lesions with a diameter ≤ 3cm.

### 2.4 Pathological examination

Tissue samples were obtained from pulmonary lesions via either transbronchial lung biopsy or CT-guided percutaneous pulmonary biopsy. The biopsy specimens were processed through chemical fixation, paraffin embedding, and special stains, followed by microscopy. Special stains include Haematoxylin–Eosin, Grocott-Gomori’s Methenamine Silver and Periodic Acid–Schiff.

### 2.5 Statistical analysis

All the statistical analyses were performed using SPSS Statistics 25 software (IBM, Armonk, NY, USA). Continuous data were described as mean ± standard deviation (SD) while categorical data were presented as count(percentage). In our study, there are many ordered categorical variables, such as baseline LFA, radiological outcome, change of LFA, and extent of lesion. For the ordered multi-classified dependent variable radiological outcome, higher assigned values indicated better outcomes. Similarly, for the ordered multi-classified variable baseline LFA, greater positivity was assigned higher values. Hence, we conducted Goodman–Kruskal gamma test, chi-square trend test (Koletsi and Pandis, 2016) and ordinal logistic regression analysis (Liangping. and Riyang., 2014; Liang et al., 2020) to explore the correlation and trends of data. The Chi-square trend test is also known as Mantel-Haenszel χ2 test or linear by Linear Test or test for Linear Trend which was used to identify the trends between two ordered categorical variables. In this study, we used it to explore the potential associations between imaging outcomes and their risk factors. The strength of correlation between the row and column variables from ordinal RxC cross-tables was assessed by the Goodman–Kruskal gamma test, or Spearman correlation analysis. Gamma test is used for the measurement of the strength of association between two ordered categorical variables (Hryniewicz, 2006). Underlying associations between LFA and radiological outcomes were evaluated using an ordinal logistic regression model and reported as odds ratios with 95% confidence intervals indicating the odds of an improved imaging outcome. All the ordinal logistic regression satisfied Brant’s test with all p values for proportional odds assumption greater than 0.05. Categorical data were compared using Chi-squared test and α segmentation was used for pairwise comparisons. Non-normally distributed continuous variables are tested via Mann-Whitney U test while normally distributed continuous variables were compared via the Student’s t-test. A p value < 0.05 was regarded as statistically significant. α segmentation was used for pairwise comparison to redefine the p-value for significance.

## 3 Results

As shown in **Table 1**, a total of 168 adult patients with PC were included in this study, of which 59.5% were male. As recommended by guidelines for pulmonary cryptococcosis, 96.4% of patients received fluconazole 400 mg daily for 6–12 months (Perfect et al., 2010; Chang et al., 2024) as the first-line treatment, while the rest were treated with voriconazole due to hematologic malignancy. The most common radiological outcome was partial absorption and the radiological remission rate was 84.5%.

**Table 1:** Demographics and clinical characteristics of participants grouped by radiological outcomes.

Characteristics	Total	Improvement group		Non-improvement group		P value
		Partial absorption	Complete absorption	Aggravated	Unchanged	
Sex						
Male	100 (59.5%)	88 (62%)		12 (46.2%)		0.131
Female	68 (40.5%)	54 (38%)		14 (53.8%)		
**Age**	52.66 ± 14.83	51.87 ± 14.8		57 ± 14.52		0.118
**BMI**	23.22 ± 3.17	23.46 ± 3.1		21.98 ± 3.29		*0.029* ^&^
Immune state						
Immunocompetent	124 (73.8%)	112 (78.9%)		12 (46.2%)		*0.000486*
Immunodeficiency	44 (26.2%)	30 (21.1%)		14 (53.8%)		
Baseline LFA						
Negative	9 (5.4%)	9 (6.3%)		0		*0.011* ^a^
Weak positive	25 (14.9%)	21 (14.8%)		4 (15.4%)		
Positive	134 (79.8%)	112 (78.9%)		22 (84.6%)		
Change of LFA						
Equal or increased	87 (51.8%)	67 (47.2%)		20 (76.9%)		*0.005*
Decreased	81 (48.2%)	75 (52.8%)		6 (23.1%)		
Symptom						
Fever	15	10		5		0.103^b^
Cough	63	55		8		0.441
Dyspnea	10	7		3		
Chest pain	11	10		1		
Coexisting disease						
Hypertension	59	47		12		
Diabetes	10	8		2		
Hyperlipoidemia	35	32		3		
Hyperuricemia	20	17		3		
CHD	8	7		1		
MST	15	12		3		
Hemopathy	7	3		4		
DTD	34	26		8		
AID	13	8		5		
CKD	6	2		4		
Mental disorders	6	4		2		
CHB	10	8		2		
**Exposure^#^**	6	6		0		

Chi-square test was used for comparison of categorical variables. ^a^P for linear-by-linear association for four-category image outcome and baseline LFA. ^b^Yates’s correction for continuity. Italics highlight the p less than 0.05. CHD, Coronary heart disease; MST, Malignant solid tumor; DTD, Digestive tract diseases mainly refer to chronic gastritis or gastroduodenal ulcer; AID, Autoimmune disease; CHB, Chronic hepatitis B. ^&^Student’s t-test. ^#^6 patients had definite past environmental exposure such as pigeon, birds or loose soil. Their pulmonary lesions were all partially absorbed after therapy.

Grouped by radiological outcome, patients with partial or complete absorption were categorized as the improvement group, while the rest were defined as the non-improvement group. Compared to the improvement group, non-improvement group had a significantly higher proportion of patients with immune deficiencies (P < 0.001) and lower BMI (P < 0.05).

When the radiological outcomes were analyzed as a four-category variable (partial absorption, complete absorption, aggravated, unchanged), both baseline LFA and changes in LFA were proved to be significantly correlated with the severity of radiological outcome. Gamma test (**Table 2**) revealed a significant negative association between baseline LFA grade and radiological outcome (γ = - 0.368, P = 0.045), indicating higher baseline LFA grades were associated with worse radiological prognosis. In contrast, patients with decreased LFA after treatment were more likely to show a better radiological prognosis, compared to those with equal or increased LFA(γ = 0.371, P = 0.012).

**Table 2:** Gamma test and trend chi-square test for radiological outcomes and potential factors.

		Image outcome				Gamma value	P value
		Aggravated	Unchanged	Partial absorption	Complete absorption		
Population		**5**	**21**	**119**	**23**		
**Immune state**	**Immunocompetent**	3 (60%)	9 (42.9%)	97 (81.5%)	15 (65.2%)	-0.241	0.167
	**Immunodeficiency**	2 (40%)	12 (57.1%)	22 (18.5%)	8 (34.8%)	-0.13^r^	0.093^h^
**Baseline LFA**	**Negative**	0	0	5 (4.2%)	4 (17.4%)	-0.368	*0.045*
	**Weak positive**	0	4 (19%)	16 (13.4%)	5 (21.7%)	-0.17^r^*	*0.011* ^h^
	**Positive**	5 (100%)	17 (81%)	98 (82.4%)	14 (60.9%)		
**Change of LFA**	**Equal or increased**	4 (80%)	16 (76.2%)	57 (47.9%)	10 (43.5%)	0.371	*0.012*
	**Decreased**	1 (20%)	5 (23.8%)	62 (52.1%)	13 (56.5%)	0.188^r^*	*0.014* ^h^
**Lesion extent**	**Single pulmonary lobe**	1 (20%)	12 (57.1%)	62 (52.1%)	11 (47.8%)	0.019	0.894
	**Unilateral lung**	1 (20%)	2 (9.5%)	20 (16.8%)	2 (8.7%)	0.011^r^	0.849^h^
	**Bilateral lungs**	3 (60%)	7 (33.3%)	37 (31.1%)	10 (43.5%)		
**Radiological morphology**	**Single nodule**	0	1 (4.8%)	5 (4.2%)	2 (8.7%)	0.027	0.828
	**Multiple nodules**	2 (40.0%)	7 (33.3%)	34 (28.6%)	7 (30.4%)	0.017^r^	0.996^h^
	**Mass**	0	0	3 (2.5%)	1 (4.3%)		
	**Pneumonia**	1 (20.0%)	10 (47.6%)	44 (37.0%)	6 (26.1%)		
	**Mixed**	2 (40.0%)	3 (14.3%)	33 (27.7%)	7 (30.4%)		
**Lesion distribution**	**Left lung**	2 (40%)	11 (52.4%)	33 (27.7%)	5 (21.7%)	0.173	0.209
	**Right lung**	0	3 (14.3%)	49 (41.2%)	8 (34.8%)	0.105^r^	0.256^h^
	**Bilateral lungs**	3 (60%)	7 (33.3%)	37 (31.1%)	10 (43.5%)		

Column percentages are shown in parentheses. ^r^coefficient of Spearman correlation analysis. *: P <0.05 for Spearman correlation test; ^h^P for linear by linear association; Italics highlight the p less than 0.05.

When the radiological outcomes were analyzed as a dichotomous variable, the proportion of patients with decreased LFA after treatment was significantly higher in the improvement group compared to the non-improvement group (χ² = 7.785, P = 0.005) (**Table 1**). The radiological findings grouped by dichotomous radiological outcomes were summarized in **Table 3**. The most common lesion extent was restricted to a single pulmonary lobe. Pneumonia was the most common radiological morphology followed by multiple nodules and mixed lesions. Quite a few participants presented cavities, bronchial inflation, halo sign, lobulation, or burr on chest CT.

**Table 3:** Radiological findings grouped by radiological outcomes.

Items	Total	Non-improvement group	Improvement group	P value
Lesion distribution				*0.01* ^d^
Left lung^A^	51 (30.4%)	13 (50%)	38 (26.8)	*0.002* (A vs B)
Right lung^B^	60 (35.7%)	3 (11.5%)	57 (40.1%)	0.031 (B vs C)
Bilateral lungs^C^	57 (33.9%)	10 (38.5%)	47 (33.1%)	0.314 (A vs C)
Lesion extent				0.876^f^, 0.756^g^
Single pulmonary lobe	86 (51.2%)	13 (50%)	73 (51.4%)	
Unilateral lung^e^	25 (14.9%)	3 (11.5%)	22 (15.5%)	
Bilateral lungs	57 (33.9%)	10 (38.5%)	47 (33.1%)	
Radiological morphology				0.849^f^, 0.504^g^
Single nodule	8 (4.8%)	1 (3.8%)	7 (4.9%)	
Multiple nodules	50 (29.8%)	9 (34.6%)	41 (28.9%)	
Mass	4 (2.4%)	0	4 (2.8%)	
Pneumonia	61 (36.3%)	11 (42.3%)	50 (35.2%)	
Mixed	45 (26.8%)	5 (19.2%)	40 (28.2%)	
**Cavity**	33	8	25	
**Lobed or sawtooth**	24	2	22	
**Halo sign**	45	5	40	
**Trachea sign**	36	2	34	

Italics indicate the significance.

^e^The lesions distributed in more than one lobe but were restricted to unilateral lung.

^f^P for Fisher test, ^g^P for Gamma test, ^d^P for Chi-square test of RxC cross-tables.

**Figure 1:**
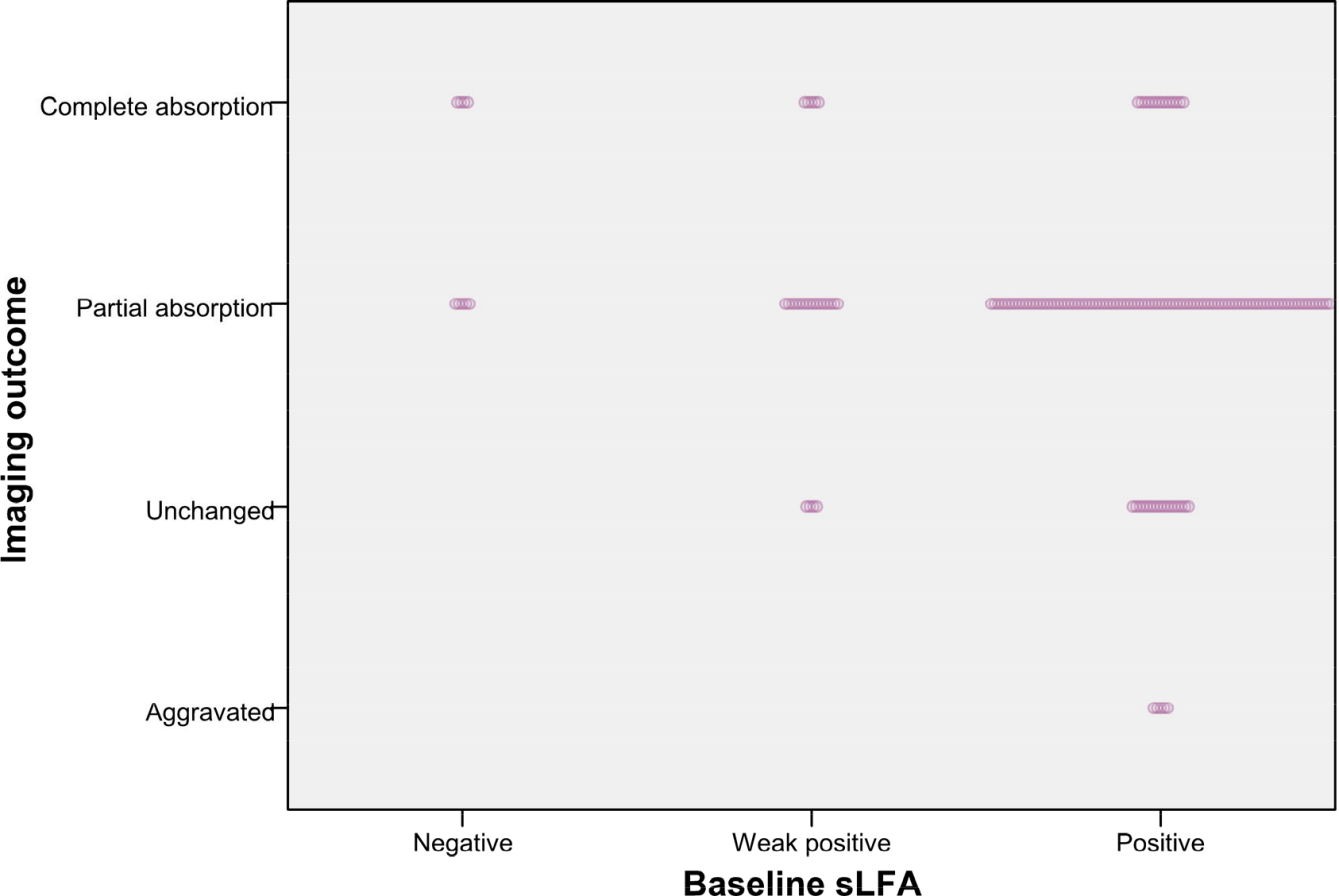
Patients with different image outcomes grouped by baseline LFA. The length of dotted band presents the number of patients.

**Figure 2:**
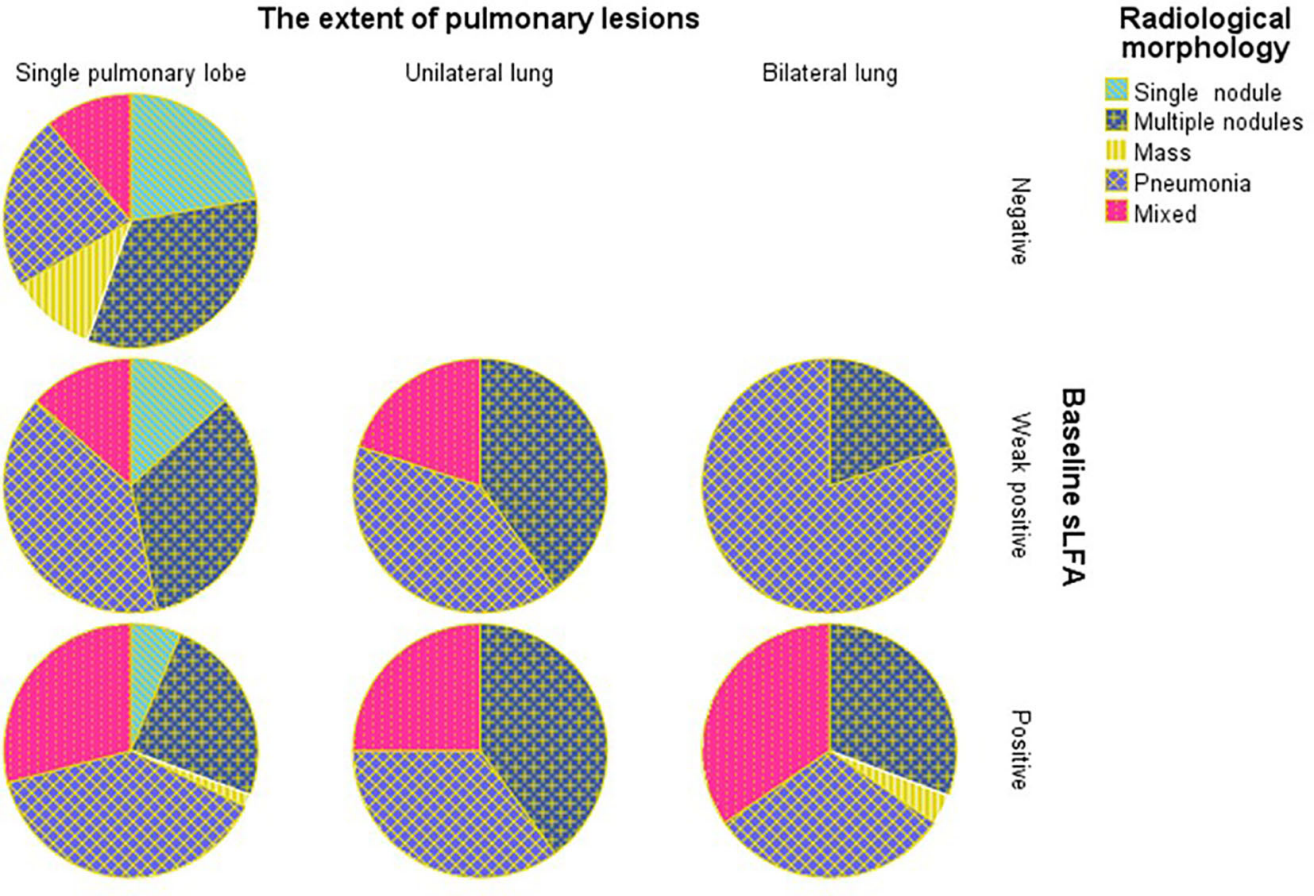
Percentage of patients with different radiological morphology grouped by baseline LFA and the extent of pulmonary lesions.

The Chi-square test of the 3x2 contingency table for the relationship between imaging outcomes and lung lesions distribution yield a p value < 0.05, prompting further pairwise comparisons to clarify the intergroup differences. Compared to lesions distributed in the left lung and bilateral lungs, those with lesions restricted to the right lung are more likely to have favorable radiological outcomes(A vs B: χ = 9.382, P = 0.002, B vs C: χ = 4.657, P = 0.031). No significant differences in radiological morphology or lesion extent were found between the two groups based on gamma test and Fisher’s test results. Notably, the distribution of baseline LFA in different imaging outcome groups was unbalanced (**Figure 1**). In the baseline LFA-negative group, no patients exhibited aggravated or unchanged imaging outcomes. All patients with aggravated image prognosis were in the baseline LFA-positive group. As the baseline LFA grade increased and the lesion distribution expanded, the proportion of mixed lesions increased, while those of single nodules decreased significantly (**Figure 2**).

**Figure 3:**
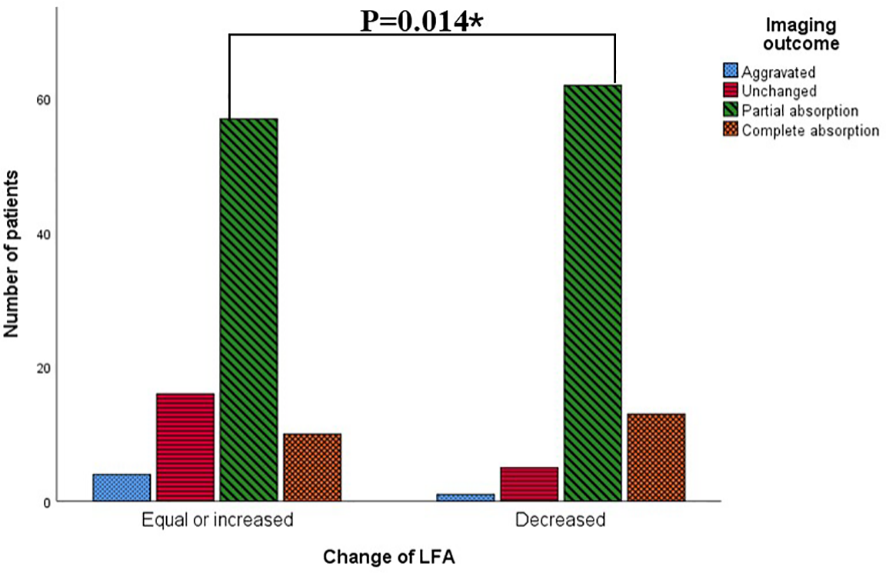
Patients with different imaging outcomes grouped by changes of LFA. *: p<0.05 by Mantel-Haenszel test.

**Figure 3** revealed that the composition ratio of imaging outcomes differed significantly between the two groups categorized by change of LFA. Patients with equal or increased LFA had a significantly higher proportion of aggravated or unchanged imaging outcomes compared to those with decreased LFA. The results of the gamma test and trend chi-square test consistently indicated that both the baseline LFA and change in LFA were significantly associated with imaging outcome (**Tables 2**, **4**). Radiological outcome tended to improve in the order of positive, weak positive, and negative baseline LFA (linear-by-linear association, P = 0.011, Spearman correlation coefficient = -0.17; γ = -0.368, P = 0.045) (**Table 2**, **Figure 1**). In addition, patients with decreased LFA after therapy were found to have significantly better imaging prognosis than those with equal or increased LFA (Linear-by-linear association, P = 0.014, Spearman correlation coefficient = 0.188; γ = 0.371, P = 0.012) (**Table 2**, **Figure 3**).

**Table 4:** Ordinal logistic regression on factors associated with radiological prognosis.

Independent variable	Categories	Estimate	SE	Wald’s χ²	P value	OR	95% CI	
							L.B.	U.B.
**Change of LFA**	Decreased	0.843	0.351	5.775	0.016*	2.324	1.168	4.623
	Equal or increased^m^	.	.	.	.	.	.	.
**Baseline LFA**	Negative	1.931	0.704	7.513	0.006**	6.895	1.734	27.424
	Weak positive	0.448	0.483	0.861	0.354	1.565	0.608	4.030
	Positive^m^	.	.	.	.	.	.	.

*P<0.05, **P<0.01. Radiological outcome was an ordinal dependent variable with four categories. OR represents the likelihood of improved imaging outcomes for the respective group compared to the control group. ^m^ Control group.

To verify the relevance between LFA and imaging outcome, we performed univariate ordinal logistic regression and found that the trend persisted. As described in **Table 4**, Patients with a decline in LFA after treatment had a higher probability of improved radiological outcomes compared to those with equal or increased LFA (OR = 2.324, P = 0.016). Patients with negative or weak positive baseline LFA had higher odds of favorable radiological outcomes than those with positive baseline LFA (OR = 6.895, P = 0.006 for LFA-negative, OR = 1.565, P = 0.006 for weak positive LFA)

## 4 Discussion

Immunocompetent patients with PC were common in China, partly due to the genetic predisposition of the Chinese Han ethnicity (Fang et al., 2015). Most previous studies focused on comparing the diagnostic efficiency of LFA and other CrAg detection methods (Liu et al., 2024). However, there have been few studies on the radiological prognosis of PC. In this study, we explored the underlying predictive factors for imaging outcomes of PC based on the retrospective analysis.

Overall, the remission rate of pulmonary lesions in PC in our cohort was 84.5% which is lower than that reported by Junyan Qu (Qu et al., 2020) but higher than the finding of Kyoung Hwa Choi (Choi et al., 2011). When the imaging outcomes were categorized as a binary dependent variable, we found significant associations between imaging outcomes and several factors, including host immune state, baseline LFA, change of LFA, and lesion distribution. Immunocompetent hosts generally showed better radiological outcomes after treatment, which was consistent with previous reports (Wang et al., 2023). Patients with lesions confined to the right lung had a higher likelihood of improved imaging outcomes, possibly due to anatomical differences between the left and right main bronchi. However, when the imaging outcomes were analyzed as a dependent variable with four categories, the significant correlation only survived in the baseline LFA and the changes in LFA based on a series of trend analyses. Thereby, baseline LFA and changes in LFA were confirmed important predictors of the imaging outcomes of PC.

We found that higher baseline LFA grades were associated with larger lesion extent, which was consistent with our previous finding (Shi et al., 2024) and a recent report (Dai et al., 2024). However, only a small quantity of studies explored the relationship between LFA and the radiological outcomes of pulmonary cryptococcosis after therapy, and none of them performed trend analysis on the correlation. In this study, we found PC patients with higher LFA level generally correlate with worse radiological outcomes. One recent study carried out by Yan-Li Zhang proves that bilateral distribution is an independent risk factor for a worse therapeutic response of PC (Zhang et al., 2024), which verifies the generalizability and rationality of our conclusions. Yi Su et al. also demonstrated that serum antigen levels were markedly elevated in patients with diffuse pulmonary infiltrates and consolidation, who generally required prolonged clinical treatment (Su et al., 2024). Early research has shown that the decrease in cryptococcal antigen titer, as detected by the LA test, lags behind radiological improvement (Kohno et al., 2015). With the advances in CrAg detection methods, LFA has been proven to have a higher detection rate for localized pulmonary cryptococcosis than the LA test (Hevey et al., 2020). However, only a few small-scale observational studies have focused on monitoring LFA titer during PC treatment. John F. Fisher et al. integrated data from several studies and discovered that decreased antigen titers were observed in 22 out of 25 patients with pulmonary cryptococcosis after treatment, although the method and timing of CrAg detection were not specified. The study conducted by Te-Yu Lin et al. also demonstrated that serum CrAg titers, detected by LA, declined during treatment for 16 patients with PC, and low serum CrAg titers predicted no relapse (Lin et al., 2009), which suggests that the CrAg titer may be a useful indicator for monitoring the therapeutic efficacy of PC.

Our study confirmed the previous findings and provided original discoveries. We adopted a case-control study design and discovered that over half of the PC patients did not experience a decrease in LFA during the treatment, which accounted for the majority of the patients with poor imaging outcomes. The chi-square test srevealed that the imaging outcomes of the LFA descending group were significantly better than those of the LFA non-descending group. This suggests that a decrease in LFA after treatment could serve as a predictive factor for radiological improvement, which has not been mentioned in previous studies. In line with the previous finding (Lin et al., 2021), we also confirmed that positive baseline LFA was a risk factor for poor radiological outcomes in PC. After antifungal therapy, the baseline LFA-negative group showed partial or complete absorption of lesions and without newly developed lesions, while the baseline LFA-positive group had a higher proportion of imaging exacerbation compared to those with weak positive LFA. The significance of correlation and trend have been validated by multiple statistical tests.

Previous studies have found an association between CrAg titer and extrapulmonary dissemination which indicated that none of the patients with serum CrAg titers ≤ 1:5 had CM (Xu et al., 2020). On the other hand, high titers of serum CrAg may suggest more extensive extra-pulmonary involvement and a worse prognosis (Hung et al., 2008). In this study, we observed that CrAg titer was also associated with intrapulmonary dissemination, which is supported by Tao Zhu et al (Zhu et al., 2018). And all lesions in the baseline LFA-negative group were confined to a single lobe, and the proportion of solitary nodule in this group was higher than that in the LFA-positive and weak positive groups.

Several strengths of this study are worth noting. We employed various statistical methods to ensure the robustness of our conclusions, which made our findings convincing. However, there are limitations to be addressed in future research. The major limitation is the use of a qualitative test for LFA instead of a quantitative test. Future research may benefit from comparing the titer of cryptococcal antigen between groups to obtain a predictive curve and make the conclusion more convincing. Besides, the small sample size in the LFA-negative group may have led to statistical bias and an unreliable result. A large-scale, multi-center study is required to validate our findings.

## 5 Conclusions

Most immunocompetent individuals with PC experience favorable imaging outcomes after antifungal therapy. A positive baseline LFA is a risk factor for poor radiological prognosis in PC, while a decrease in LFA after treatment with azole antifungals predicts lesion resorption Our findings provide new insights into the evaluation of PC prognosis as well as clinical decision-making and treatment protocols. Clinicians need to pay more attention to PC patients with positive or high-titer baseline LFA or persistent positive LFA during therapy since they may have unsatisfactory therapeutic response and radiological prognosis. It is necessary to closely monitor their LFA and chest imaging and make timely adjustments to therapeutic strategy as needed.

## Data Availability

The raw data supporting the conclusions of this article will be made available by the authors, without undue reservation.
